# Long-Term Competitive Dynamics of Two Cryptic Rotifer Species: Diapause and Fluctuating Conditions

**DOI:** 10.1371/journal.pone.0124406

**Published:** 2015-04-16

**Authors:** Carmen Gabaldón, María José Carmona, Javier Montero-Pau, Manuel Serra

**Affiliations:** Institut Cavanilles de Biodiversitat i Biologia Evolutiva, Universitat de València, Valencia, Spain; University of Girona, SPAIN

## Abstract

Life-history traits may have an important role in promoting species coexistence. However, the complexity of certain life cycles makes it difficult to draw conclusions about the conditions for coexistence or exclusion based on the study of short-term competitive dynamics. *Brachionus plicatilis* and *B*. *manjavacas*are two cryptic rotifer species co-occurring in many lakes on the Iberian Peninsula. They have a complex life cycle in which cyclical parthenogenesis occurs with diapausing stages being the result of sexual reproduction. *B*. *plicatilis* and *B*. *manjavacas*are identical in morphology and size, their biotic niches are broadly overlapping, and they have similar competitive abilities. However, the species differ in life-history traits involving sexual reproduction and diapause, and respond differently to salinity and temperature. As in the case of certain other species that are extremely similar in morphology, a fluctuating environment are considered to be important for their coexistence. We studied the long-term competitive dynamics of *B*. *plicatilis* and *B*. *manjavacas* under different salinity regimes (constant and fluctuating). Moreover, we focused on the dynamics of the diapausing egg bank to explore how the outcome of the entire life cycle of these rotifers can work to mediate stable coexistence. We demonstrated that these species do not coexist under constant-salinity environment, as the outcome of competition is affected by the level of salinity—at low salinity, *B*. *plicatilis* excluded *B*. *manjavacas*, and the opposite outcome occurred at high salinity. Competitive dynamics under fluctuating salinity showed that the dominance of one species over the other also tended to fluctuate. The duration of co-occurrence of these species was favoured by salinity fluctuation and perhaps by the existence of a diapausing egg bank. Stable coexistence was not found in our system, which suggests that other factors or other salinity fluctuation patterns might act as stabilizing processes in the wild.

## Introduction

Competitive theory predicts that the strength of competition involving two or more species is greater between closely related species than between distantly related species [1]. According to the phylogenetic limiting similarity hypothesis [2], cryptic species—which have a close phylogenetic relationship and high morphological similarity—are expected to have similar ecological requirements (i.e., niche conservatism) [2–5] and, hence, to experience stronger competition and competitive exclusion [6]. However, there are many examples of co-occurrence of cryptic species (e.g., [7–10]). Coexistence may be mediated by subtle niche differentiation, e.g., differential susceptibility to predation and/or resource partitioning [11–13]. In certain cases, this niche differentiation is based on small morphological differences that were neglected due to the past taxonomic status of the species (e.g.,[14]). Moreover, coexistence of cryptic species would be favoured as a result of their shared features if these features translate into similar fitness. Similar fitness (an equalizing process) implies that weak stabilizing processes (niche differentiation) can be sufficient for stable coexistence [15,16].

The potential role of the organism’s life cycle in promoting the coexistence of competitors has been demonstrated [17,18]. For instance, investment in diapause by a superior competitor may provide an opportunity for coexistence to inferior ones [19]. Moreover, the occurrence of life cycle stages relatively free of competition—as are diapausing stages—is a necessary condition for the storage effect, a stable coexistence process based on environmental fluctuations [16–20]. A fluctuating environment may allow species coexistence if conditions favour different species at different times, indefinitely delaying the outcome of competitive exclusion.

In complex life cycles, the study of competitive dynamics and conditions for coexistence or exclusion becomes more difficult as fitness components relate to fitness in a complex way. This makes it difficult to predict the outcome of competition solely from observations of short-term dynamics such as somatic growth in plants or clonal proliferation in cyclical parthenogens. This problem might particularly affect congeneric species because their life cycle strategies may diverge [21,22].

Cyclically parthenogenetic rotifers have complex life cycles involving sexual and asexual reproduction. This is the case of *Brachionus plicatilis* and *B*. *manjavacas*, two cryptic species that belong to the *B*. *plicatilis* species complex. Their species status is guaranteed by prezygotic reproductive isolation which has been confirmed by mating experiments as well as by the lack of evidence for hybridization in field studies [23,24]. These two species co-occur in salt ponds of the Iberian Peninsula [25,26], with marked salinity fluctuations. In temperate climates, rotifer populations are active in the water column during a fraction of the year (the so-called growing season) even in permanent ponds. Thus, they periodically re-colonize the water column from banks of diapausing eggs, which are the product of sexual reproduction. During the growing season, their populations can fluctuate due to episodic investment in sex or due to exposure to substantial environmental variation. *B*. *plicatilis* and *B*. *manjavacas* have identical morphology and size [27,28] and consistently do not show ecological differentiation in biotic niche axes[29]. Hence, because these species inhabit a rather spatially homogeneous habitat, classical niche differentiation mechanisms that operate independently of environmental fluctuations [16,30], such as resource partitioning or differential susceptibility to predation, are unlikely to influence their coexistence [29].

*B*. *plicatilis* and *B*. *manjavacas* differ in life-history traits [31], such as their growth response to salinity and temperature [32], and their patterns of investment in diapause and hatching of diapausing eggs [31]. Single-species cultures have shown that the salinity tolerance ranges of the two species largely overlap [32]. However, if salinity is low, *B*. *plicatilis* shows better performance and invests more in the production of diapausing eggs than *B*. *manjavacas*, even at the cost of decreasing its current proliferation rate. Diapausing eggs of *B*. *plicatilis* also show a greater viability than those of *B*. *manjavacas*. Therefore, although *B*. *manjavacas* is predicted to be a better competitor (i.e., can tolerate a wide range of salinities), a growing season of low salinity might offer an opportunity window for *B*. *plicatilis* to produce the diapausing eggs needed for its persistence.

Previous empirical studies have analysed the importance of competition among coexisting cryptic species of zooplankters and the conditions to avoid exclusion (e.g., [13,32–38]). Several of these studies emphasise the importance of changing environments and of disturbance for the maintenance of coexistence [13,34,35,37,39]. However, empirical evidence on the implications of fluctuations is still scarce. When dealing with changing environments, zooplankters have to face fluctuations of different magnitude scales: those fluctuations leading alternation between suitable and unsuitable periods, and those fluctuations causing variation in conditions when the habitat is suitable. The long-term competitive output will rely on the rates of diapausing-stages production under a range of suitable conditions, as diapausing stages are the way to survive during unsuitable periods. The objective of the present work was to study experimentally the long-term competitive outcome of *B*. *plicatilis* and *B*. *manjavacas*. According with the habitat characteristics of these species we explored the effect of salinity fluctuations, comparing constant salinity and different patterns of time-varying salinity. In our experiments, periods of habitat unsuitability were simulated experimentally. In this way, investment in diapause had a role on the competitive experimental dynamics. Hence, it included the whole life cycle of these rotifers mimicking what is expected to occur in nature. Like in the wild, each suitable period the active population was initiated by the hatchlings of diapausing eggs produced in previous active growth periods (i.e., growing seasons). Two conditions for diapause duration were simulated. In one, no diapausing egg was allowed to survive longer that a single unsuitable period. Similarly to the terminology used for plant seed banks, this pattern was called ‘without diapausing egg bank’. In the other condition, called ‘with diapausing egg bank’, survival of diapausing eggs for longer than a single unsuitable period might occur [40]. Our approach to the experimental study of zooplankton is novel. As far as we are concerned, it is the first time that competitive dynamic of two zooplanktonic species is explored not only during the growth of the active populations in the water column but also taking into account the effect of the production of diapausing eggs which remain in the sediment and are able to hatch in next growing seasons. Notice also that a putative stable species coexistence could rely on diapause.

We are interested in testing the following points. First, we hypothesise that the dynamics of competition depends on salinity; specifically the expectation is that low salinity favours *B*. *plicatilis* whereas high salinity is advantageous to *B*. *manjavacas*. This hypothesis is based on previous results on single-species cultures, and, if accepted, it would show the predictive power of single-species studies on the outcome of competition. Second, we want to know whether the divergence in diapause investment of these species [31,41], makes possible stable coexistence or, at least, delays exclusion. Third, we hypothesise that fluctuating salinity affects the competitive outcome, we expect coexistence or, at least, an extremely long time to exclusion.

## Materials and Methods

### Rotifer clone foundation and maintenance

Diapausing eggs were isolated from sediment samples collected in Salobrejo Lake (Eastern Spain: 38° 54.765' N, 1° 28.275' W) as described in Gabaldón et al. [29]. Permission for the field work was issued by the Junta de Comunidades de Castilla-La Mancha, Spain (Consejería de Agricultura y Medio Ambiente).Clones of *B*. *plicatilis* and *B*. *manjavacas* were established by asexual proliferation of the hatchlings from these diapausing eggs. Each clonal line was identified to the species level by PCR-RFLP [29]. For each rotifer species, 25 clones were founded and kept individually as stock cultures in 15 mL flasks at 25°C and a salinity of 10 g/L. Each week, one-half of each stock culture was renovated with fresh medium. This medium was f/2-enriched saline water [42] prepared with commercial sea salt (Instant Ocean; Aquarium Systems) in which the microalga *Tetraselmis suecica* (Collection of Marine Microalgae of Marine Sciences Institute from Andalusia, Spain) was grown as a source of food for the rotifers. Microalgae were grown at 10 g/L salinity, 25°C, and constant aeration and illumination (PAR: approx. 35 μmol photons m^-2^ s^-1^). The pre-experimental and experimental rotifer culture media were the same as described for the stock cultures, except for salinity (see below).

### Experiment 1: Interspecific competition and different salinities

Experimental populations of *B*. *plicatilis* and *B*. *manjavacas* were grown in competition for 24 day cycles in the lab. Field observations show that such short growing seasons can be common and are enough to observe both species in the water column in Salobrejo pond [32]. Moreover, due to the short generation time of *Brachionus* females, this period is enough for diapausing egg hatching, to reach population density for sexual reproduction initiation, and to produce diapausing eggs (e.g.[40]). The successive growing seasons were restarted from the hatchlings of diapausing eggs produced in the previous season, mimicking the process that occurs in a natural pond after desiccation and refilling. The experiment lasted for six cycles (growing seasons) or less if one species was not detected in the diapausing eggs produced in two consecutive cycles or no diapausing egg was observed after a cycle.

These competition experiments were performed under five different salinity regimes: (1) 10 g/L constant salinity (all growing seasons at 10 g/L); (2) 40 g/L constant salinity (all growing seasons at 40 g/L); (3) 10–40 g/L alternating salinity (first growing season at 10 g/L); (4) 40–10 g/L alternating salinity (first growing season at 40 g/L); and (5) 10 to 40 g/L increasing salinity within each growing season. Thus, the experiment consisted of 15 cultures (five salinity regimes x three replicates).

Before the experiments, pre-experimental cultures of each clonal line were started from the stock cultures and grown in culture medium at 10 and 40 g/L in 75 mL flasks (2 rotifer species x 2 salinities x 25 clonal lines = 100 pre-experimental cultures). Each clonal line was reared in exponential growth for at least three generations to control for maternal effects (e.g., [43]) and to acclimate the rotifers to the experimental conditions. Juvenile females from these pre-experimental cultures were used to initiate experimental cultures. For each experimental culture (i.e., salinity regime and replicate), 10 juvenile females were randomly selected from each of the 25 clones of the two rotifer species and inoculated in 500 mL of culture medium containing 250,000 cells/mL of *T*. *suecica*. Experimental cultures were kept in 2000 mL plastic containers at 25°C in darkness and under constant agitation (60 rpm). Darkness was selected in order to avoid algae proliferation. Every six days, 500 mL of fresh culture medium containing 250,000 cells/mL of *T*. *suecica* at 10 or 40 g/L was added to each experimental culture. Thus, three feeding events within growing season were performed, and the final volume at the end of the growing season was 2000 mL. In the case of the fifth salinity regime (10 to 40 g/L increasing salinity), the salinity was increased in each feeding event, first at 20 g/L, then at 30 g/L and finally at 40 g/L.

At the end of every 24 day cycle (i.e., growing season), rotifer populations were filtered through 30 μm Nitex mesh, and the diapausing eggs produced were collected and isolated in Petri dishes that were exposed at 25°C to allow water evaporation. Dried diapausing eggs were kept at 4°C and in the dark for 28 days to ensure the completion of the obligate period of dormancy of *Brachionus* diapausing eggs [44]. Then, one-half of these diapausing eggs were allocated to species identification, and the other half of the eggs were used to restart the following growing season. Immediately before the restart of a new growing season, the dried diapausing eggs were rehydrated and incubated for 72 hours at 25°C and constant illumination to induce hatching [45,46]. To improve hatching [31], induction salinity conditions were set at 5 g/L for those eggs starting the next growing season at 10 g/L and at 20 g/L for those starting at 40 g/L. All hatchlings were used to initiate the new growing season. Because *B*. *plicatilis* has an extended diapausing egg hatching pattern [31], the remaining unhatched diapausing eggs were monitored daily, and the new hatchlings were successively added to the experimental cultures during the first six days of each growing season (i.e., until the cultures were fed for the second time). The diapausing eggs remaining unhatched after this time were discarded.

Species identification of the harvested eggs at the end of each growing season was performed by PCR-RFLP as detailed in Gabaldón et al.,[29]. In this case, DNA was extracted from individual diapausing eggs. For each replicate, up to 100 diapausing eggs were identified if possible. This identification allowed the proportion of diapausing eggs produced by *B*. *plicatilis* and *B*. *manjavacas* during the competition dynamics to be determined. For each regime, the analyses were performed until we determined that all the identified individuals belonged to the same species during two consecutive growing seasons. This information allowed us to assume that the other species had been excluded.

### Experiment 2: Interspecific competition, fluctuating salinity, and diapausing egg bank

We used a design similar to that in Experiment 1 to test the effect of salinity fluctuations on the competitive dynamics between *B*. *plicatilis* and *B*. *manjavacas* under two different conditions for diapause duration (1) “without diapausing egg bank” as in Experiment 1 and (2) “with diapausing egg bank”. In this second experimental condition, in contrast to Experiment 1, the successive growing cycles were started with all of the diapausing eggs harvested in the previous season (i.e., non-hatched diapausing eggs were not discarded but were inoculated in the culture). As a result, recruitment from the diapausing eggs might occur at any time during the 24 days of a growing season (i.e., delayed hatching can take place). Additionally, the harvested eggs in a growing season might have not been produced in that growing season (i.e., eggs that could persist in diapause for longer than a single growing season are included). These are the conditions that characterize a diapausing egg bank [40], as new clones can originate from diapausing eggs produced in any of the previous growing seasons. Three replicates of each condition were used (2 conditions x 3 replicates = 6 experimental cultures). Experimental culture populations were founded with hatchlings from diapausing eggs formed during Experiment 1 after single-species cultures had been achieved. For each replicate, 500 diapausing eggs of each species were mixed in a Petri dish with 50 mL of saline water at 10 g/L and allowed to hatch for 72 hours at 25°C and constant illumination. We selected the salinity regime of 10–40 g/L alternating salinity because Experiment 1 showed that a salinity fluctuation starting at low salinity favours *B*. *plicatilis*, which was commonly inferior competitor. The experiment ended when one of the two species was not detected in the diapausing eggs produced after two consecutive growing seasons.

To avoid fluctuations in food supply, each growing season was carried out in a chemostat with 950 mL of culture medium and a dilution rate of 0.15 day^-1^. The inflow rotifer culture medium consisted of *T*. *suecica* continuously cultured in f/2 medium (see above) at 25°C and constant illumination.

During each growing season, the effluent volume from each experimental rotifer culture was recovered in a container with a filter of 30 μm Nitex mesh. This mesh size ensured that individuals and eggs were retained. Every 1 or 2 days, the material retained on the filters was transferred to Petri dishes with saline water, and the diapausing eggs were counted. These eggs were then dehydrated and kept at 4°C in darkness until the start of the next growing season. After 24 days (the duration of the growing season), the rotifer populations were filtered, and diapausing eggs were isolated, dehydrated and kept at 4°C in the dark for 28 days.

As in the first experiment, the next growing season was initiated after 28 days of diapause from hydrated diapausing eggs from the previous growing season, and one-half of the eggs were allowed to hatch. To obtain a successful hatching, the hatching salinity was 10 g/L if the next season was at 10 g/L and 20 g/L if it was at 40 g/L. The other half of the diapausing eggs were used for species identification based on PCR-RFLP. At least 100 diapausing eggs per growing season were identified.

### Statistical analysis

Heterogeneity among replicates within a cycle and salinity condition was tested by chi-square test (counts of diapausing eggs identified as *B*. *plicatilis* and *B*. *manjavacas* x replicate). The association between the competitive output (winner species) in Experiment 1 and salinity regime (10 g/L constant, 40 g/L constant, fluctuating salinity) was tested by a chi-square test with Yate’s correction for continuity. This analysis could not be performed for Experiment 2 due to low count number. In these tests, performed with R statistical software v. 2.12.1 [47], p-values were compute by Monte Carlo simulation.

In order to assess the effect of salinity on the diapausing egg production of competitors, we computed a rate of diapausing egg increase (log of diapausing eggs produced/eggs inoculated per growing season) for each replicate where both species were presented. The arithmetic difference between the rates of the competitors was used as the dependent variable in an ANOVA with salinity (10 g/L vs. 40 g/L) as factor. These analyses were carried out using SPSS statistical software [48].

## Results

**Fig 1:**
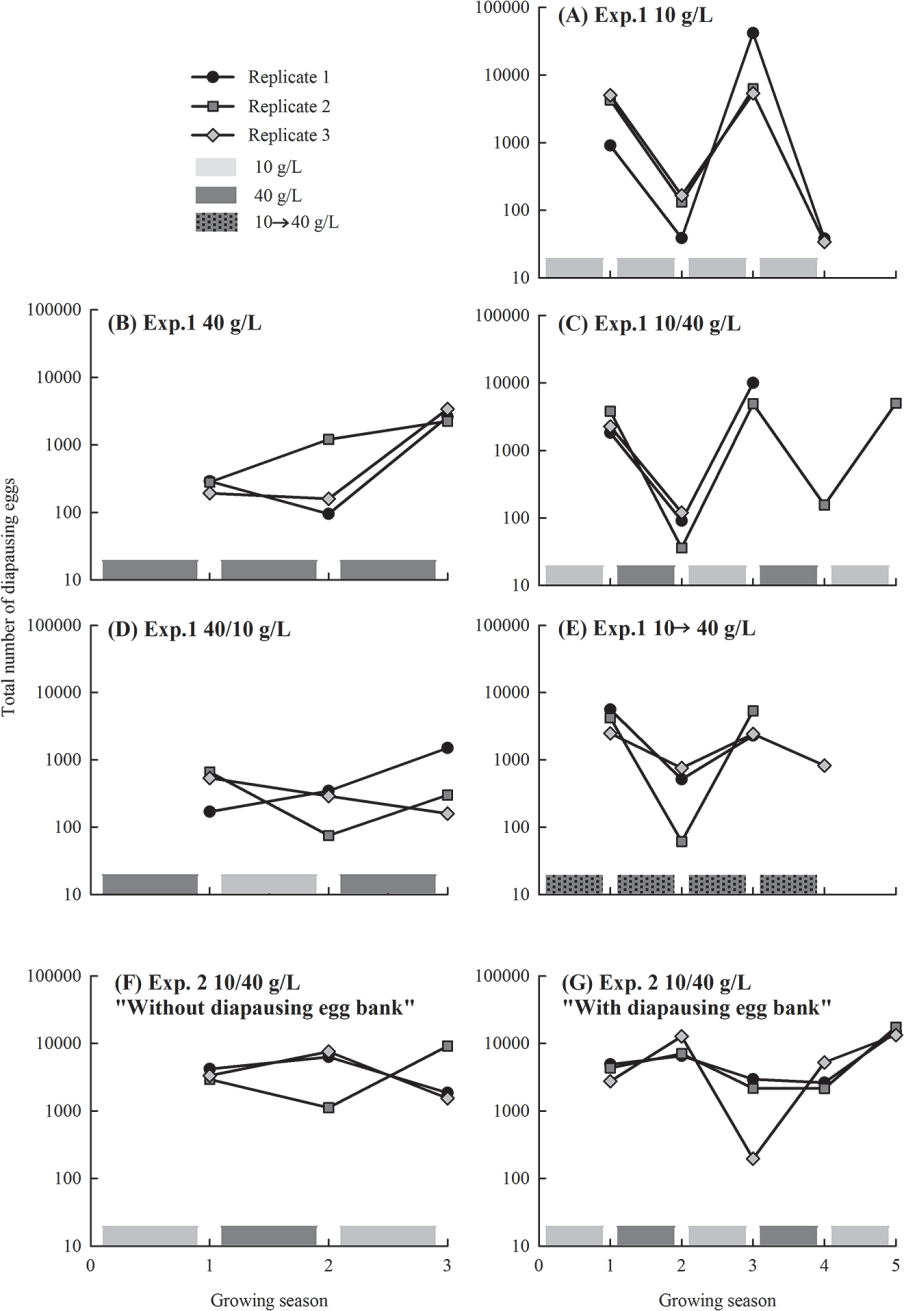
Total number of diapausing eggs produced during each growing season in response to the salinity fluctuation regime in both experiments. Experiment 1 (A) 10 g/L constant salinity (all growing seasons at 10 g/L); (B) 40 g/L constant salinity (all growing seasons at 40 g/L); (C) 10–40 g/L alternating salinity (the first growing season at 10 g/L followed by the second growing season at 40 g/L, and then again at 10 g/L, and so on); (D) 40–10 g/L alternating salinity (the same as (C) but starting at 40 g/L); and (E) 10 to 40 g/L increasing salinity (each growing season started at 10 g/L, but the salinity was gradually increased during the growing season until it reached 40 g/L); and Experiment 2 (F-G) 10–40 g/L alternating salinity regime in two conditions: without and with diapausing egg bank.

**Fig 2:**
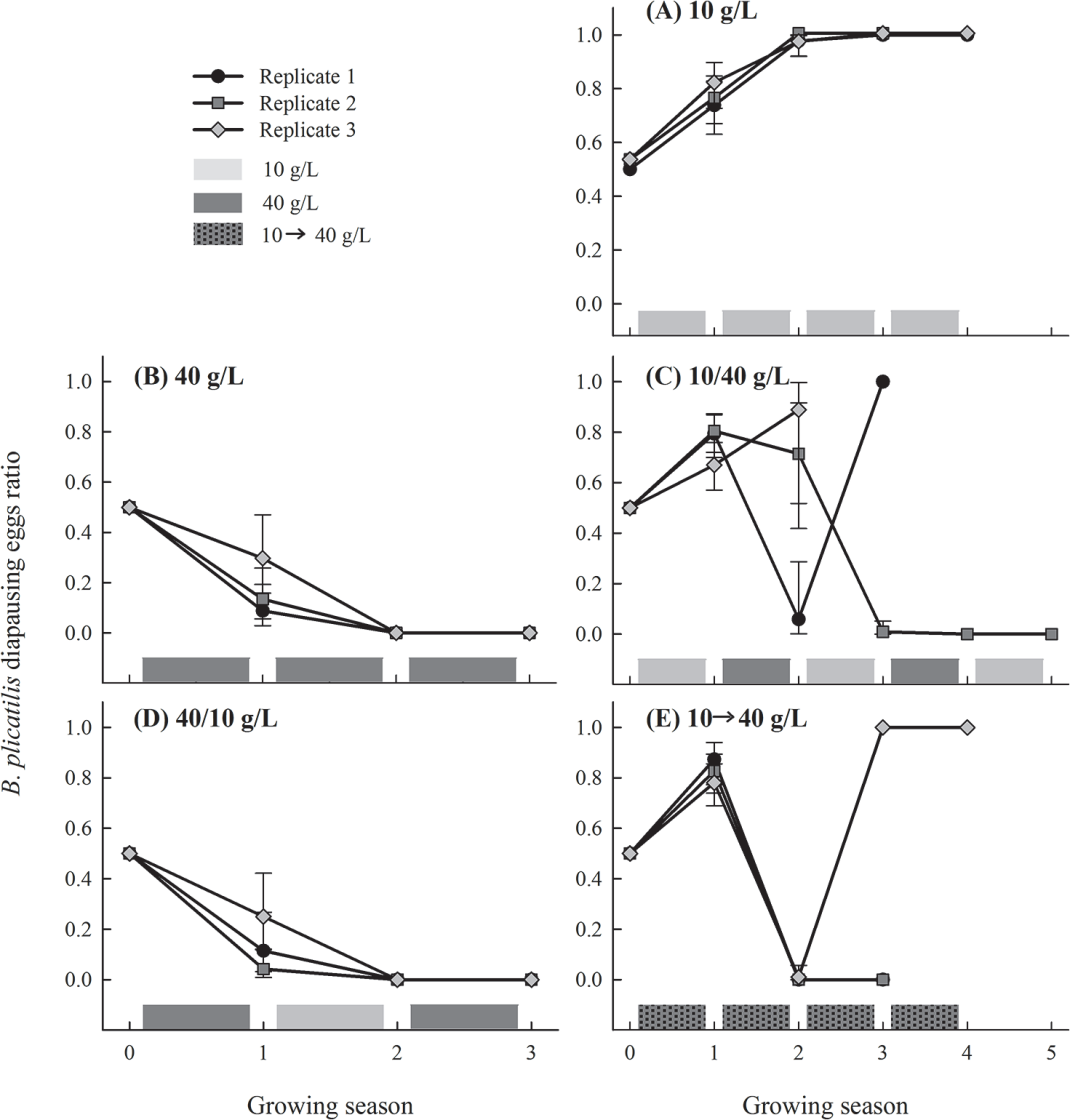
*B*. *plicatilis* diapausing egg ratio in the egg bank produced during each growing season in response to the salinity fluctuation regime in Experiment 1. (A) 10 g/L constant salinity (all growing seasons at 10 g/L); (B) 40 g/L constant salinity (all growing seasons at 40 g/L); (C) 10–40 g/L alternating salinity (the first growing season at 10 g/L followed by the second growing season at 40 g/L, and then again at 10 g/L, and so on); (D) 40–10 g/L alternating salinity (the same as (C) but starting at 40 g/L); and (E) 10 to 40 g/L increasing salinity (each growing season started at 10 g/L, but the salinity was gradually increased during the growing season until it reached 40 g/L). Vertical bars are ± SE.

In Experiment 1, the diapausing egg number averaged 1870 per growing period and replicate (n = 50; range: 0–3,970) (Fig 1). The total number of diapausing eggs identified as *B*. *plicatilis* or *B*. *manjavacas* via molecular methods was 3,738. We followed an average of 3.27 cycles per replicate. This relatively low number of cycles was due primarily to the absence of one of the species in the diapausing eggs produced during two consecutive growing seasons. However, one of the replicates at 10–40 g/L alternating salinity was lost in its fourth cycle due to unknown causes after a 100% frequency of *B*. *plicatilis* frequency had been achieved, and another replicate maintained at the same conditions did not produce diapausing eggs. The proportion of *B*. *plicatilis* in the diapausing eggs produced after each growing season in response to the five salinity fluctuation regimes (Experiment 1) is shown in Fig 2. If the salinity regime was constant throughout the growing seasons, one of the species was excluded in all replicates in a consistent way. At a lower salinity, *B*. *manjavacas* was excluded; at a high salinity, *B*. *plicatilis* was excluded. *B*. *plicatilis* was also excluded under a fluctuating-salinity regime in which the salinity of the first growing season was high (40–10 g/L treatment).

In the fluctuating salinity regime starting at low salinity and in the regime of increasing salinity during the growing season, the same species was not consistently excluded in the three replicates. In certain cases, we found that a species became extinct even after its competitor was extinct. In these cases, extinction occurred after a substantial decrease in the production of diapausing eggs. The competitive output was significantly associated to the salinity regime (*X* ^*2*^ = 6.96; *d*.*f*. = 2; *p*-value = 0.03).

**Fig 3:**
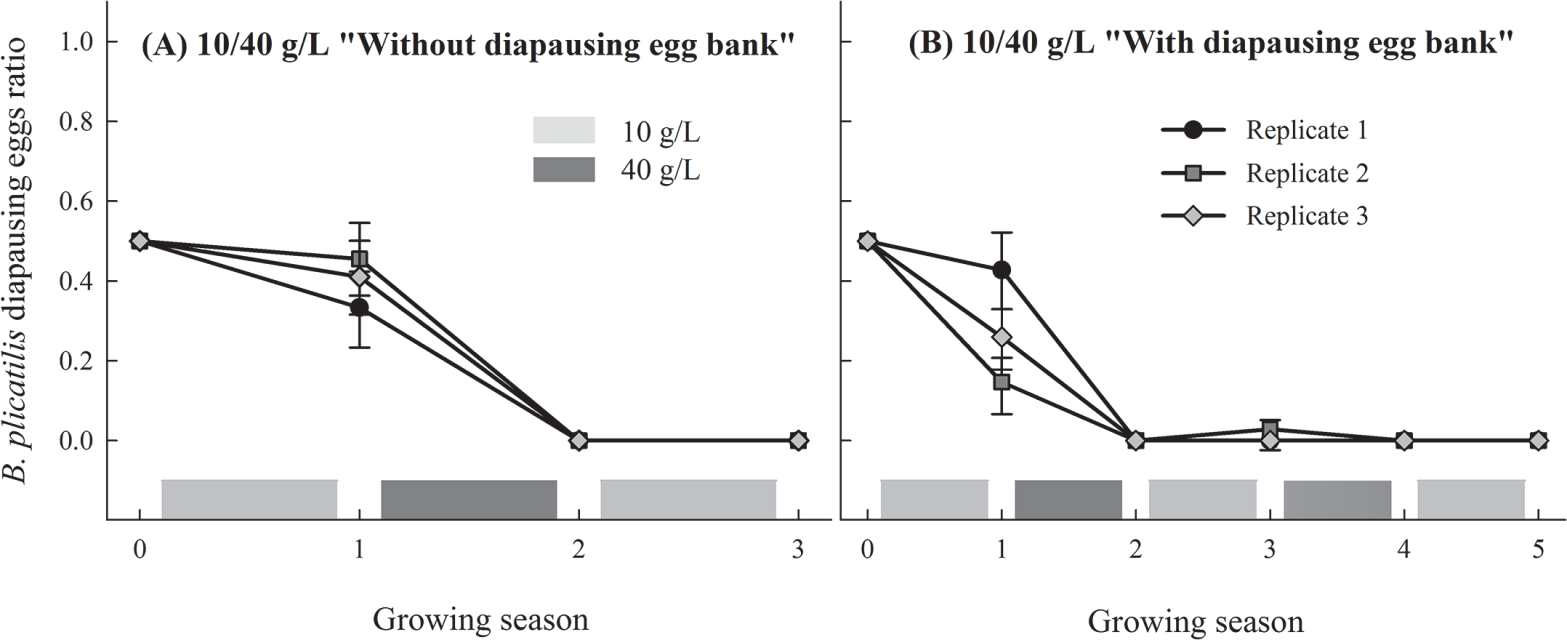
*B*. *plicatilis* diapausing egg ratio in the egg bank harvested after each growing season in Experiment 2. Rotifer populations were grown under a 10–40 g/L alternating salinity regime in two conditions (A) without diapausing egg bank and (B) with diapausing egg bank. Vertical bars are ± SE.

In Experiment 2, the diapausing egg number averaged 5,717 per growing season and replicate (*n* = 24; range: 197–17,457) (Fig 1). A total of 2,601 diapausing eggs were identified by molecular methods as *B*. *plicatilis* or *B*. *manjavacas*, and an average of four cycles per replicate were followed. The population densities were noticeably higher. No anomalies (e.g., a lack of diapausing egg production) occurred. Fig 3 shows the proportion of *B*. *plicatilis* in the diapausing eggs after each growing season in response to a fluctuating salinity regime in the two conditions tested (with and without a diapausing egg bank). *B*. *plicatilis* was excluded by *B*. *manjavacas* in all replicates of both conditions. However, in one of the replicates of the condition “with diapausing egg bank” *B*. *plicatilis* was able to recover—62 diapausing eggs were observed—in the third growing season, which corresponded to one of the low salinity periods (10 g/L) within the alternating salinity regime.

The replicates in each growing season, were frequently heterogeneous in their proportion of diapausing eggs belonging to each species. According to chi-squared test, heterogeneity was significant (p-value< 0.05) in seven out of the thirteen cases where the test could be applied.

The difference (*B*. *manjavacas* minus *B*. *plicatilis*) in the log rate of increase of diapausing eggs per growing season average 1.48 and 3.2 at 40 g/L salinity in Experiment 1 and 2 respectively, showing an advantage for *B*. *manjavacas*. The corresponding averages were -0.91 and -1.18 at 10 g/L. The effect of salinity on this difference was significant (*F* = 21.8; *d*.*f*. = 1,29; *p*-value< 0.01).

## Discussion

*B*. *plicatilis* and *B*. *manjavacas* are a well-documented instance of the long-term coexistence of cryptic species [25,26], which is a more common phenomenon than previously thought. These species also provide a good example of how close phylogenetic relationship, especially if associated with an almost total morphological similarity, translates into niche overlap, particularly on the biotic axis [29]. However, the two species physiologically respond differently to temperature and salinity [31], two factors with low spatial variation but with large temporal fluctuation in their habitat [49]. Additionally, each species has evolved different patterns for the trade-off involved in their complex life cycles. *B*. *plicatilis*, as an opportunistic species, invests earlier in diapause, producing diapausing eggs with a higher viability and a more extended hatching pattern than *B*. *manjavacas* [31]. In this study, we have proved that salinity affects the competitive outcome of *B*. *plicatilis* and *B*. *manjavacas*. The observed outcomes in constant salinity support our prediction made from single-species cultures where species performance was assessed [31,32]: at low constant salinity *B*. *manjavacas* is excluded whereas at high constant salinity *B*. *plicatilis* is excluded. Competition experiments between zooplankters have commonly focused on the dynamics of interactions in the water column. In contrast, our experiment integrated the complete life cycle of the competitors from clonal propagation to sex initiation and the production of diapausing eggs, and from the production to the hatching of these eggs. This approach allowed us to explore whether exclusion in the water column implies competitive exclusion as a result of the long-term dynamics. Note that the merely observation of exclusion in the water column is not conclusive, as the excluded competitor could have sufficient time to produce a large number of diapausing eggs before its exclusion in the water column. As expected, our results show that exclusion occurs rapidly in constant salinity, in 2–3 growing seasons, despite the similar performance of both species when they grow without competitor. Interestingly, the prediction that *B*. *plicatilis* was superior to *B*. *manjavacas* at low salinity has been demonstrated in our experiments. This prediction was based not only on the former species growth rate, but also on its potential to invest in diapause at this salinity level. Previous works have reported that the potential growth rate (*r*_*pot*_; [50], [31,41]), which is a measure of performance that takes into account the growth rate and the diversion of resources to diapause, was higher in *B*. *plicatilis* than in *B*. *manjavacas* at low salinity. Therefore, our results support that the higher performance of *B*. *plicatilis* at low salinity is used to produce diapausing eggs at the cost of decreasing its rate of asexual proliferation, i.e., its within-growing season growth rate. This strategy would cause the demographic dominance of *B*. *plicatilis* at low salinity in a sequence of growing seasons. Our results suggest that the dynamics of competition in the water column, if based solely on the rates of proliferation, could not be a good predictor of the long-term output because the competitor that was in the process of being excluded from the water column might be contributing more diapausing eggs in the sediment bank.

Under fluctuating salinity, our results showed that the dominance of one species over the other also tended to fluctuate. The fluctuations in the relative frequencies of the species were consistent with the species-specific preferences in terms of salinity: at low salinity, the recovery of *B*. *plicatilis* was more probable than at high salinity, which is consistent with the effect of salinity showed by ANOVA. Moreover, Experiment 1 showed that, on average, salinity fluctuations tended to delay exclusion by 0.6 growing seasons. Salinity fluctuation within a growing season had not a noticeably different effect when compared to salinity fluctuation among growing seasons. Under fluctuating conditions, *B*. *manjavacas* tended to be the persistent species. This finding is consistent with the observation that its performance was superior to that of *B*. *plicatilis* at intermediate fixed salinities in single-species cultures [31,32]. Although exclusion was observed in all the replicates, the persistent species varied among replicates within the same fluctuation regime. This heterogeneity is not surprising given the similar fitness of both species [31].

The saline ponds where these rotifers co-occur in the Iberian Peninsula are habitats characterised by substantial salinity fluctuations within and between growing seasons [32,49]. Hence, coexistence or, at least, an extremely long time to exclusion might be expected in our fluctuating experimental conditions, but these outcomes did not occur. The lack of long co-occurrence periods in our experiments can be a result of several factors. First, co-occurrence in the wild might be maintained by spatial, among-pond heterogeneity. Second, the fluctuation regime in our experiments might not be the same process that stabilizes coexistence in the wild. Other factors (correlated or not correlated with salinity) or other salinity fluctuation patterns might act as stabilizing processes. However, this seems to be unlikely because, the experimental salinity does have an effect on the species excluded and because the experimental salinity fluctuation range was observed in the natural habitats where the species occur [31,32] and both species tolerate the extreme values in this range. Third, demographic stochasticity can play a role in preventing coexistence. Notice that the number of diapausing eggs fluctuated strongly over growing seasons, the number being rather low in some of them. This explanation is suggested by the heterogeneity among replicates and the variation in the outcome of competition in two of the experimental conditions. Additionally, we found a few replicates in which a population became extinct after the other species had already been excluded. Moreover, variation among replicates was higher in Experiment 1, where pulses in the food supply and the corresponding changes in population densities were expected to increase intrinsic stochasticity. Contrasting to experimental populations, natural rotifer populations are composed of extremely large numbers of individuals. For instance, a density up to 8 females/mL has been reported for the *B*. *plicatilis* population of Salobrejo pond [32] which considering the pond dimensions [51] allow to roughly estimate a population size of 230 x 10^9^ individuals for this species. Hence natural populations will have much lower demographic stochasticity. If so, small volume experiments could yield exclusion for conditions allowing coexistence in nature.

Experiment 2 incorporates a diapausing egg bank which involves the opportunity of delayed hatchlings of diapausing eggs produced in previous seasons to take part in the competitive dynamics. In nature, not all diapausing eggs produced in a growing season hatch when the conditions are favourable for population growth, instead they accumulate in the sediment forming diapausing egg banks [52,53]. The recovery of the outcompeted *B*. *plicatilis* only happened in one of the replicates carried out “with diapausing egg bank”. This constitutes a weak evidence of the buffering effect of the diapausing egg bank, but it suggests that exclusion of *B*. *plicatilis* is more difficult when a bank is present as occur in natural habitats. Interestingly, this strategy agrees with the fact that diapausing eggs of *B*. *plicatilis* have lower degradation rates than those of *B*. *manjavacas* [31], allowing them to overcome longer unsuitable periods.

This study is the first to empirically address the competitive dynamics of the cryptic species *B*. *plicatilis* and *B*. *manjavacas* by growing the two species together. It is also the first pair-wise competition experiment in rotifers that includes the whole life cycle. We have demonstrated that these competitors are unable to coexist in a constant environment. Their competitive capabilities are similar, and the result of the competitive dynamics between the two species is dependent on the salinity regime. We found that *B*. *plicatilis* has higher productivity in terms of diapausing eggs produced when it grows at 10 g/L, showing a faster growth at low salinity. The timespan of co-occurrence of these competing species increases due to salinity fluctuation and perhaps due to the existence of a diapausing egg bank. However, none of these effects is dramatic, and stable coexistence has not been found. We suggest that demographic stochasticity, which is also associated with fluctuations and is important in small scale experiments, is obscuring a stabilizing the effect of fluctuations based on niche differentiation.
